# Social Cognition in Adolescents with Developmental Language Disorder (DLD): Evidence from the Social Attribution Task

**DOI:** 10.1007/s10803-022-05698-6

**Published:** 2022-08-15

**Authors:** Claire L. Forrest, Vanessa Lloyd-Esenkaya, Jenny L. Gibson, Michelle C. St Clair

**Affiliations:** 1 Department of Psychology and Human Development, IOE, UCL’s Faculty of Education and Society, 25 Woburn Square, London, WC1H 0AA UK; 2 Department of Psychology, University of Bath, Bath, UK; 3 Faculty of Education, University of Cambridge, Cambridge, UK

**Keywords:** Developmental language disorder, Social cognition, Theory of mind, Adolescents, Social and emotional difficulties

## Abstract

Social cognition impairments may explain social, emotional and behavioural difficulties (SEBD) in individuals with Developmental Language Disorder (DLD). In a novel approach, the Social Attribution Task (SAT) was used to examine this association. SAT narratives were coded from 53 participants [n = 26 DLD; n = 27 typical language development (TLD)] matched on age (*M*_*age*_ = 13;6) and gender (35.9% female). Parents reported SEBD. Adolescents with DLD performed worse than their TLD peers on the majority of SAT indices and had higher peer (*d* = 1.09) and emotional problems (*d* = .75). There was no association between social cognition abilities and SEBD. These exploratory findings suggest social cognition should be further examined in this population.

**Supplementary Information:**

The online version contains supplementary material available at 10.1007/s10803-022-05698-6.

Developmental language disorder (DLD) affects approximately 7% of the population, presenting as a difficulty with expressive and/or receptive language that cannot be accounted for by any other cognitive impairment or neurodevelopmental condition (Norbury et al., 2016). Research has shown that this population is more at risk for additional social and emotional problems, with increased rates of anxiety, depression and victimisation compared to their typically developing (TD) peers (Conti-Ramsden & Botting, 2008; van den Bedem et al., 2018). What is less clear, however, is how the relationship between DLD and poor socioemotional outcomes manifests. One suggestion is that there may be a comorbid social cognition deficit responsible for negative outcomes such as social skills difficulties (Bishop, 1997). However, with the exception of Botting and Conti-Ramsden (2008), most research on social cognition in the DLD population has focused on children. Given that findings from longitudinal studies indicate an increase in peer problems in adolescents with DLD (St Clair et al., 2011), and evidence from the general population indicates that social cognition is still developing in adolescence (Blakemore & Choudhury, 2006), it is important to continue examining social cognition abilities in the DLD population throughout childhood and adolescence. To do this, the current study employs the Social Attribution Task (Heider & Simmel, 1944), which has not been used previously in the DLD population.

Social cognition can be broadly defined as the ability to process and understand social interactions, such as attending to others and interpreting social cues. In order to respond appropriately in social situations, individuals must draw on their ability to interpret others’ thoughts, feelings and motives, also known as Theory of Mind (ToM; Premack & Woodruff, 1978) or ‘mentalizing’ (Frith & Frith, 2003). These abilities are highly correlated with language abilities (Dunn et al., 1991); therefore, individuals with a language difficulty may be at a disadvantage in this domain. Indeed, a recent meta-analysis demonstrates that children with DLD have lower social cognition abilities than their TD peers (Nilsson & de Lopez, 2016). Vissers and Koolen (2016) proposed three causal models to explain the relation between language and Theory of Mind (ToM) in children with DLD. Namely, early experiences of ToM such as joint attention predict language growth; language skills predict later ToM abilities or there is another factor (e.g. working memory) driving both abilities simultaneously. While the exact causal pathway is unknown, social cognition is important to study as it may explain the increased social difficulties that children and adolescents with DLD are known to experience (Smit et al., 2019; Vissers & Koolen, 2016). Specifically, individuals with DLD have deficits in social skills such as initiating a conversation (Brinton et al., 1997) and resolving conflicts (Bakopoulou & Dockrell, 2016; Marton et al., 2005), which require awareness and understanding of others’ mental states.

Recent research has examined impaired social cognition as a predictor of the social problems experienced by individuals with DLD. For example, children with DLD performed worse than their TD peers on emotion recognition tasks and hypothetical social scenarios, which was significantly associated with higher teacher-rated socioemotional problems (Bakopoulou & Dockrell, 2016). Similarly, poor performance on the Strange Stories task was associated with more peer problems and lower scores on a friendship and social activities scale in adolescents with DLD but not their TD peers (Botting & Conti-Ramsden, 2008). Furthermore, poor performance on false belief (FB) tasks such as “Unexpected Contents” and the “Change of Location” task among children with DLD predicted more “dislike” sociometric ratings from their classmates (Andres-Roqueta et al., 2016).

Despite the established relationship between social cognition and social outcomes (Dunn & Cutting, 1999), there are considerable methodological issues that arise when examining social cognition. As social cognition is an umbrella term it is difficult to measure all aspects of this concept, and many studies rely on FB tasks which are not entirely reflective of the skills required in daily social interactions. This is exemplified by the numerous children with autism who are able to pass these tasks but who still experience difficulty navigating social situations in their day-to-day life (Abell et al., 2000). Additionally, the instructions for the Change of Location task (or “Sally-Anne task”) place a high demand on receptive language skills, which may not be appropriate for assessing social cognition in children and young people with language difficulties. For instance, Miller (2001) demonstrated that children with DLD performed similarly to their chronologically-age-matched peers on the Sally-Anne task when the language demands were low, but their ability to pass the task was similar to language-age-matched controls when the instructions imposed a greater linguistic load. It is important to consider the appropriateness of the task when testing social cognition in individuals with DLD in order to ensure the task is measuring social cognition and not language ability.

Other studies have used visual tasks such as emotion labelling of photographs to demonstrate poor theory of mind abilities in children with DLD (Bakopoulou & Dockrell, 2016), although the static nature of this task does not reflect real-life social situations. Tasks that are more interactive and involve social judgements and decision making may provide research findings that are more reflective of children’s lived experiences and more impactful for parents. There are also varying findings depending on the design of the task. For example, there was no group difference in emotion recognition between children with DLD and their TD peers on tasks using cartoon characters instead of photographs (Ford & Milosky, 2003; McCabe & Meller, 2004). Nevertheless, Ford and Milosky (2003) found that children with DLD performed worse than their TD peers when asked to identify emotions using contextual cues. However, this could be a result of the verbal demands of the task as there was no language-age-matched control group for comparison.

A final critique of the literature is that many studies investigating social cognition in individuals with DLD have focused on children. While Bakopoulou and Dockrell (2016) sampled an older group aged 8–11 years and Farmer (2000) had one group of participants with a mean age of 11, only one study has examined social cognition in an adolescent sample. Botting and Conti-Ramsden (2008) examined performance on the “Eyes Task” (Baron-Cohen et al., 2001a) and the “Strange Stories” task (Happé, 1994) in a group of 16-year-olds with and without DLD. They found that adolescents with DLD performed worse on the social cognition tasks than their TD peers and poorer social cognition abilities were more closely associated with poorer social outcomes in the DLD group (Botting & Conti-Ramsden, 2008). The lack of research into social cognition abilities among adolescents with DLD is somewhat concerning given that DLD is a pervasive condition with long-term effects. Children do not “grow out of” DLD but maintain their reduced language ability in comparison to peers throughout development (Conti-Ramsden et al., 2012; Norbury et al., 2017). More specifically, there are increased peer problems in adolescence (St Clair et al., 2011). This increase is in line with findings from the general population that peer relations become much more important during adolescence, with social functioning influencing mental health outcomes (Geoffroy et al., 2018; van Harmelen et al., 2017). Although FB tasks are usually passed by age 4 for TD children and age 7 for autistic children (Frith & Frith, 2003), there is evidence to suggest that adolescents are still not as good as adults at ToM tasks, such as perspective taking (Symeonidou et al., 2016). The idea that social cognition is still developing in adolescence is corroborated by brain imaging studies that show different areas of the brain are involved at different ages (Blakemore & Choudhury, 2006). Therefore, it is necessary to conduct more research during this time period using tasks that are appropriate for adolescents and for the DLD population.

## Current Study

The current study compared the Social Attribution Task (SAT) performance of adolescents with DLD to their peers with typical language development (TLD). The SAT consists of a silent animation of simple line drawings of a rectangle, large triangle, small triangle and small circle which participants are asked to describe. The original study found that adults would attribute emotions and behaviours to the shapes in the short film clip, usually describing the big triangle as bullying the smaller triangle and circle (Heider & Simmel, 1944). Since then, it has been used to compare the social cognitive abilities of children, young people and adults with autism spectrum disorders (ASD) in comparison to their typically developing peers (Abell et al., 2000; Klin, 2000; Klin & Jones, 2006). To the authors’ knowledge, this is the first study to use the SAT with participants with DLD and we believe it is an appropriate task to investigate social cognition in this population. This task places a very low demand on verbal comprehension as the participant is simply asked to describe what they see after watching a silent animation of moving shapes. The video format of the SAT provides a more interactive and engaging measure of social cognition than typical ToM tasks and is suitable for all ages. Indeed, the SAT could be argued to be more accessible than other ToM tasks as it provides more data about participants’ understanding of social information than the Eyes Task (Baron-Cohen et al., 2001a) but does not pose as heavy a load on receptive language abilities as hypothetical scenarios such as the Strange Stories task (Happé, 1994). In contrast to the dichotomous scoring of typical ‘pass/fail’ FB tasks, the SAT has more opportunities to be correct and therefore provides a broader measure of social understanding (Klin, 2000). In particular, the Animation Index is scored based on the level of social attribution from each category, such as behaviours, perceptions, emotions, relationships, etc., that the participant mentions when describing the scene, not the frequency of each specific word (see supplementary materials (S1) for further details). Therefore, participants are judged on the quality of social attribution in their response and are not penalised for giving a shorter answer, which may be expected from individuals with a language difficulty.

The aim of the current study was to determine whether there are group differences in social cognition abilities between adolescents with a history of DLD and their age-and-sex-matched peers (TLD group). Social cognition abilities were measured by performance on five indices: Animation Index (attributing social meaning to the animation); Person Index (describing the shapes as people); Salience Index (identifying key social features of the animation); Theory of Mind (ToM)—Affective Index (ascribing emotional terms to the shapes) and ToM—Cognition Index (ascribing mental state terms to the shapes). Secondly, the study aimed to investigate whether performance on the SAT is related to peer and emotional problems, as measured by the parent-rated Strengths and Difficulties Questionnaire (SDQ; Goodman, 1997). It was predicted that participants with DLD would perform worse across all the SAT indices than their TLD age-and-sex-matched peers. That is, participants with DLD would score lower in their ability to attribute social meaning to the animation and in their descriptions of shapes as people. In addition, participants with DLD were expected to mention fewer key social points (showing a poorer understanding of the story) and use fewer cognitive and affective mental state terms than the TLD group when describing the actions of the shapes (demonstrating poorer social cognition). It was also predicted that adolescents with DLD would receive higher parent-ratings of peer and emotional problems than the TLD group and that these difficulties would be predicted by performance on the SAT. Associations between language ability and each of the indices were also explored to determine what extent performance on the SAT was influenced by language skills.

## Methods

### Ethics

Ethical approval was granted by the University of Bath Psychology Ethics Committee (Ref: 15-245).

### Recruitment

**Fig. 1:**
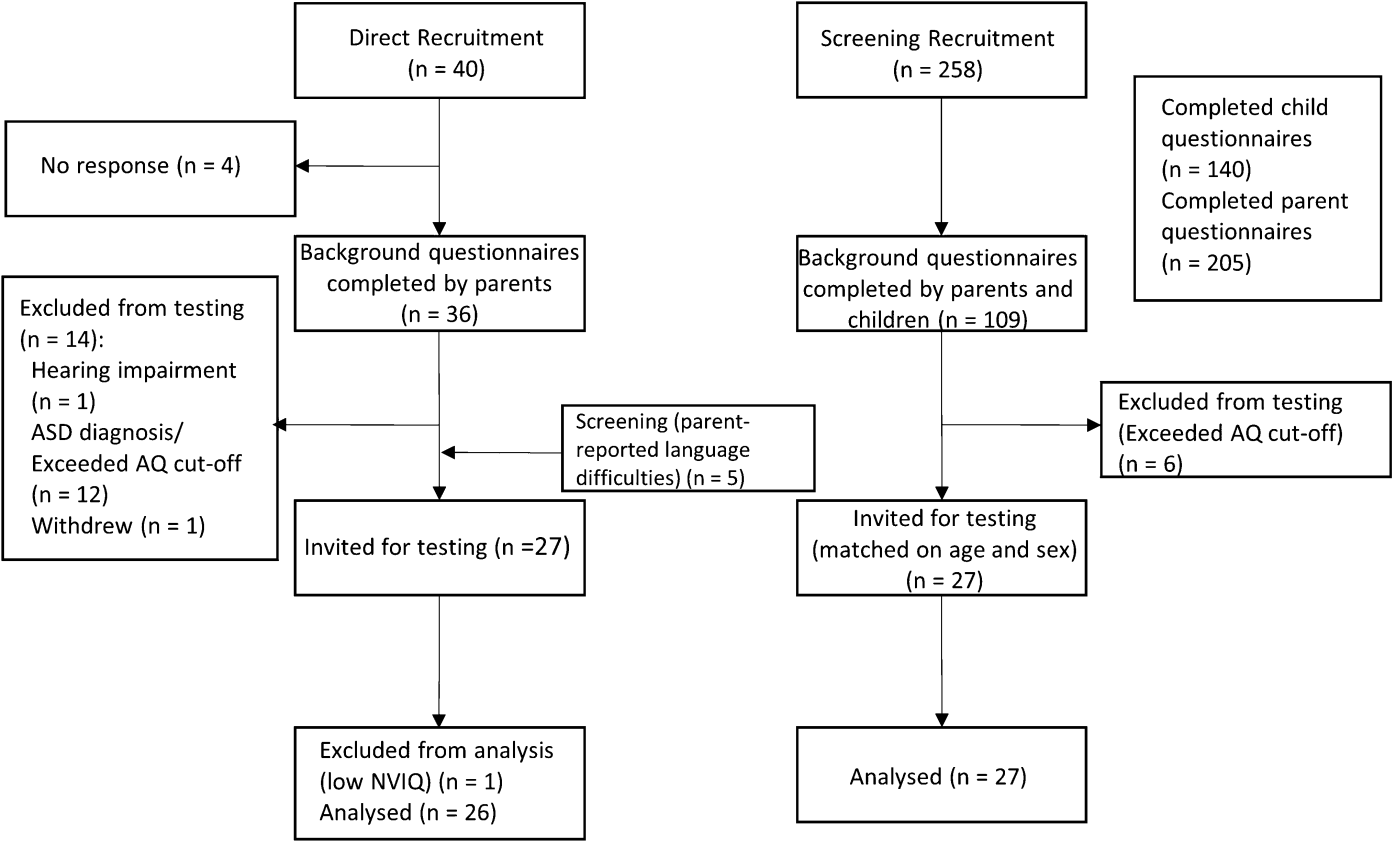
Recruitment flowchart

There were two recruitment streams for the study (see Fig. 1). Participants with a diagnosis of DLD were recruited by referral from professionals and online support groups. In addition, the TLD comparison group were identified via flyers displayed locally or on social media and were screened for suitability. Any participants with a history of language difficulties identified in the TLD group were included in the DLD group. Participants were all secondary school students aged 11–18 years old and were native English speakers. All participants attended mainstream secondary schools, although three participants were recruited from a specialised language unit within a mainstream school. Exclusionary criteria for the study consisted of parent-report of hearing impairments, intellectual disabilities and diagnoses of autism spectrum disorder (ASD). Symptoms of ASD were particularly important to screen out because poor social cognition is one of the main characteristics of ASD and adolescents with these symptoms may have confounded our findings on social cognition in DLD. Therefore, in addition to parent report of ASD diagnosis parents also reported on their children’s social skills using the Autism Quotient (AQ; Baron-Cohen et al., 2006) while participants aged 16 years and over completed the self-report version (Baron-Cohen et al., 2001b). Participants who exceeded the AQ cut-off were not eligible for the study and a low score (more than 2 SD below the mean) on the nonverbal IQ measure in the testing phase provided further exclusionary criteria.

Participants were included in the DLD group if they had a history of DLD. Participants were recruited either through referrals from a local speech and language therapy service employed by the local authority to provide services to schools, referrals from Special Educational Needs Coordinators (SENCos) within schools, or from flyers posted in online support groups for DLD. A screening procedure, consisting of a background questionnaire, language scale and socioemotional measures (see section “Participants” for details) was used to recruit TLD participants matched on age (within 6 months) and sex, as well as additional DLD participants. Of the 258 screening packs sent to participants, 109 were completed by both parent and participant.

Forty participants with a diagnosis of DLD were initially identified for the study by direct referral. Five participants with parent report of language difficulties or with a low score on the self-report measure (CC-SR) identified through the screening procedure were included in the DLD group. Their inclusion in the DLD group was corroborated by low expressive and/or receptive language subtest scores (see section “Clinical Evaluation Language Functioning—Fourth UK Edition (CELF-4^UK^)” for details). Four participants did not respond to invitation emails. One participant was not eligible due to a hearing impairment, twelve participants were excluded due to a diagnosis of autism or exceeding the cut-off on the AQ and one participant withdrew from the study.

Twenty-seven participants were invited to the testing stage as part of the DLD group, with twenty-seven matched controls identified via the screening process forming the TLD group matched on age (within 6 months) and sex. One further participant was excluded from the DLD group after scoring more than 2 SDs below the mean on the nonverbal IQ measure. This was to ensure that the DLD group consisted of participants with language difficulties but not intellectual difficulties. This resulted in a total sample of 26 adolescents with DLD and 27 TD participants matched on age and sex.

### Participants

The total sample had an average age of 13 years and 6 months (*SE* = 2.26 months) and approximately 36% were female (see Table 1 for details). English was the only language spoken at home in the majority of cases, although one participant in the DLD group spoke a second language and two participants in the TLD group spoke a second language. As expected, the DLD group were significantly more delayed in speech and language development compared to the TLD group. They were also more delayed in reaching early self-help milestones compared to the TLD group as reported in the background questionnaire. Level of parental education differed significantly between the two groups, with more parents in the TLD group completing postgraduate studies compared to the DLD group. Additionally, the DLD group had a significantly lower socioeconomic status as measured by the Income Deprivation Affecting Children Index (IDACI) Rank. In the current sample, the IDACI Rank ranged from 303 to 32,662 in the DLD group and from 13,021 to 32,489 in the TLD group, with an overall group mean of 22,388.47 (*SE* = 1030.57). On average, the TLD group consisted of individuals from less deprived areas than the DLD group.

**Table 1:** Demographics of Developmental Language Disorder (DLD) group, Typical Language Development (TLD) group and the whole sample

	DLD (n = 26)	TLD (n = 27)	Total (n = 53)	DLD vs TLD
Mean age in years; months (SE months)	13;6 (3.15)	13;6 (3.30)	13;6 (2.26)	–
Female %	34.6	37	35.9	*X*^2^ .03
Mean IDACI rank (SE)^a^	19,602.17 (1640.43)	24,865.19 (1113.82)	22,388.47 (1030.57)	− 6.79 (− 1.21, − 1.42)*
Language spoken				*X*^2^ .31
English only %	96.2	92.6	94.3	
English plus other %	3.8	7.4	5.7	
Motor development				.49 (.15, 1.59)^^^
Delayed %	26.9	3.7	15.01	
Typical %	53.8	81.5	67.9	
Fast %	19.2	14.8	17	
Speech and language development				.05 (.01, .21)^^^***
Delayed %	69.2	7.4	37.7	
Typical %	26.9	70.4	49.1	
Fast %	3.8	22.2	13.2	
Self-help development				.17 (.05, .63)^^^**
Delayed %	46.2	3.7	24.5	
Typical %	42.3	85.2	64.2	
Fast %	11.5	11.1	11.3	
Biological parents				*X*^2^ 2.25
Yes %	92.0	100	96.2	
No—adopted %	8.0	0	3.8	
Parental marital status				3.85 (.89, 16.55)^^^
Married %	68.0	88.9	78.8	
Separated %	16.0	7.4	11.5	
Divorced %	16.0	3.7	9.6	
Parental psychological distress^b^				*X*^2^ .01
Yes %	16.0	14.8	15.4	
No %	84.0	85.2	84.6	
Parental education				.19 (.06, .58)^^^**
Secondary school %	44.0	18.5	30.8	
Diploma %	12.0	0	5.8	
Undergraduate degree %	36.0	44.4	40.4	
Postgraduate degree %	8.0	37	23.1	

Statistics are b coefficients or odds ration where marked ^ (95% confidence interval) and chi square where marked *X*^2^. Regressions for age and IDACI Rank were performed on transformed data

*p < .05, **p < .01, ***p < .001

^a^n = 51

^b^As measured by endorsement of suspected or diagnosed mental health difficulties in background questionnaire

### Measures

#### Questionnaires for Direct Recruitment and Screening Samples

##### Background Questionnaire

This was completed by the parent/carer of the participant and consisted of seventeen questions regarding the child’s early development, academic history, physical/mental health history and family mental health history. Questions included the speed at which developmental milestones in language, motor skills and self-help were met and whether the child had any learning difficulties (suspected or diagnosed). Postcode information was also obtained to provide a measure of socioeconomic status, the Income Deprivation Affecting Children Index (IDACI) Rank (Smith et al., 2015). The IDACI Rank is based on the percentage of children living in families that are income deprived in Lower-layer Super Output Areas (LSOAs) across England, where 1 = most deprived neighbourhood and 32,844 = least deprived neighbourhood. School postcodes were used when home postcodes were missing (n = 5) and two participants did not have either information available.

##### The Strengths and Difficulties Questionnaire (SDQ)

The SDQ (Goodman, 1997) was completed by the participant’s parent/carer. This questionnaire consists of 25 items which form five scales (Peer Problems; Emotional Problems; Hyperactivity; Conduct Problems and Prosocial scale), the first four of which are totalled to produce the Total Difficulties score. The SDQ is a well-established measure and has a test–retest reliability of .85 (Goodman, 1999). The scales of interest were the Emotional Problems and Peer Problems subscales, each consisting of five items rated on a scale of *Not True* (0), *Somewhat True* (*1*) and *Certainly True* (*2*). Total scores for each subscale range from 0 to 10.

##### The Autism Spectrum Quotient (AQ)

The adolescent version of the AQ was completed by parents of children aged 12–15 years old (Baron-Cohen et al., 2006), while the adult version was completed by participants aged 16 years or over (Baron-Cohen et al., 2001b). Both scales consist of 50 items referring to the domains of: social skills (e.g. “I prefer to do things with others rather than on my own”); attention switching (e.g. “I prefer to do things the same way over and over again”); attention to detail (e.g. “I often notice small sounds when others do not”); communication (e.g. “Other people frequently tell me that what I’ve said is impolite, even though I think it is polite”) and imagination (e.g. “I find making up stories easy”). Items are rated as ‘Definitely agree’, ‘Slightly agree’, ‘Slightly disagree’ or ‘Definitely disagree’ and responses that endorse autistic-like behaviours are scored 1 point. A sum score of 30 or more on the parent-report, or 32 or more on the self-report is classified as a cut-off for ASD symptoms. Participants exceeding these cut-offs were not eligible for the current study.

#### Questionnaire for Screening Sample

##### The Communication Checklist Self-report (CC-SR)

The CC-SR (Bishop et al., 2009) was completed by the participant. This questionnaire consists of 70 questions about communication abilities. The participant rates the items on a scale of 0—Less than once a week (or never); 1—About once a week; 2—Once or twice a day or 3—Several times a day (or all the time). These items form three composite scales. The Structural Language composite describes aspects of language such as grammar and meaning. For example, “I mix up ‘he’, ‘she’, ‘it’ and ‘they’” and “I use short sentences”. The Pragmatic Skills composite contains items relating to language use in social contexts. For instance, “People tell me I talk too much” and “I give detailed information when a more general comment would be fine”. Finally, the Social Engagement composite is comprised of items regarding nonverbal communication and social functioning. For example, “I feel anxious when I am with other people” and “I find it hard to know when people are upset or annoyed”. Positive items are reverse scored and a scaled score lower than 5 on the Structural Language composite and greater than 7 on the Pragmatic Skills composite is indicative of DLD (*M* = 10, *SD* = 3). Internal consistency for each of the composites is greater than .85 (Bishop et al., 2009).

#### Assessment

##### Clinical Evaluation Language Functioning—Fourth UK Edition (CELF-4^UK^)

In order to measure language ability, two subtests from the CELF-4^UK^ (Semel et al., 2006) were administered. The Recalling Sentences subtest requires participants to listen to sentences of increasing length and complexity and repeat verbatim, providing a measure of expressive language. The Word Classes—Receptive subtest requires participants to pick two words out of a list of four that are best matched, providing a measure of receptive language ability in the current study. Both subtests have an excellent rating of reliability with an internal consistency coefficient of .92 and .91 respectively (Semel et al., 2006).

##### Wechsler Intelligence Scale for Children—Fourth UK Edition (WISC-IV^UK^)

The Block Design subtest was administered to provide a measure of nonverbal ability (Wechsler, 2004). This task requires participants to use 3D blocks to recreate 2D patterns of increasing complexity. Block Design is a measure of spatial awareness and contributes to fluid reasoning. One participant scoring more than 2 *SD* below the mean (*M* = 10, *SD* = 3) was dropped from analysis. Additionally, any participants with parent-report of cognitive problems from the background questionnaire were not eligible for the study.

##### Social Attribution Task (SAT)

**Fig. 2:**
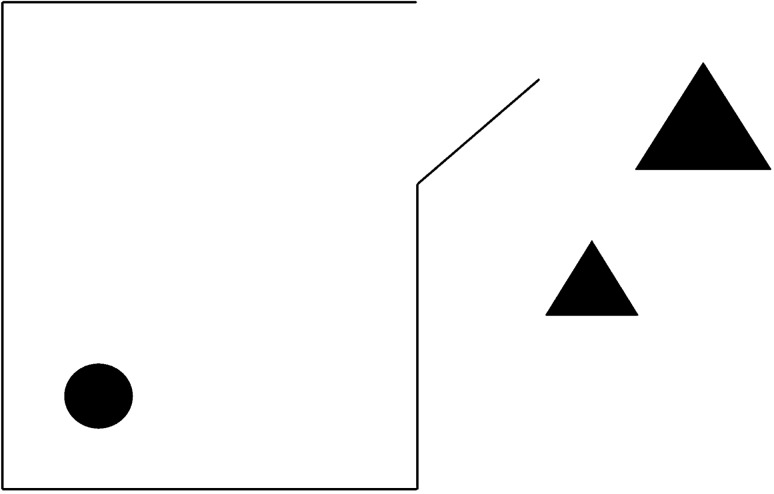
Example of a scene from the social attribution task (SAT) video. Recreated with permission from Heider and Simmel (1944)

The SAT (Klin, 2000) was administered to participants on a laptop. Participants watched a silent video of three animated shapes (large triangle, small triangle, and small circle), lasting 1 min 16 s (Fig. 2). They were asked, “What happened in the video clip?”, answering as completely as they can (narrative 1). Next they were shown the same animation separated into six shorter clips and asked, “What happened here?” after each one (narratives 2–7). Finally, the participants were asked to think of the shapes as people (if they had not already done so) and describe “What kind of person is the… (big triangle/little triangle/circle)?” (narratives 8–10). Examples of responses from participants in each group are provided in the supplementary materials (S2). The number of words used in all ten narratives was recorded, as was the number of independent clauses (‘T-Units’). The following indices were examined in this study: *Salience Index*; *Animation Index*; *Theory of Mind (ToM)—Cognition Index*; *ToM—Affective Index*; *Person Index*. The first four indices are scored from narratives 1–7, while the Person Index uses narratives 8–10. The *Salience Index* provides a measure of the proportion of the twenty key social features that are most often noticed by typically developed participants (e.g. stating that there are three agents (big triangle, small triangle and circle), observing the direction of hostility is from the large triangle towards the small triangle and circle, noticing that the circle hides because it is afraid, etc.). Each response that matches the scoring criteria is awarded one point out of a possible twenty and converted to a percentage score. The *Animation Index* measures the participant’s ability to attribute social meaning on a scale of 0–6, ranging from no social attribution to very high levels of social attribution. The *ToM—Cognition Index* states the proportion of T-Units containing cognitive mental state terms (e.g. wants, hiding, tries, bullying, etc.). The *ToM—Affective Index* provides a measure of the proportion of T-Units containing emotional terms (e.g. scared, angry, celebrating, jealous, etc.). Finally, the *Person Index* measures the participant’s ability to ascribe psychological properties to the shapes on a scale of 0–9, where 0 indicates no response and 9 indicates human characteristics for each of the three shapes. See supplementary materials (S1) for further details.

### Procedure

Informed consent and assent was obtained from parents/carers and participants. Parents/carers in the DLD group completed the consent form, background questionnaire, AQ and SDQ online or returned the forms in a freepost envelope. Participants from the DLD group were then invited to the assessment stage in a quiet room either at the University, their school or their home. Participants who were recruited through schools completed informed assent forms and were screened with the CC-SR while their parents/carers gave informed consent and completed the AQ, SDQ and an abridged version of the background questionnaire, either online or via paper copies. Any participants from this screening process who met criteria for the DLD profile on the CC-SR or who received reports of language difficulties in the background questionnaire were invited to the assessment stage and included in the DLD group. Those that had no language difficulties were matched on age (within 6 months) and sex to form the TLD group. Again, the assessment stage was completed wherever was convenient for the participant. Parents/carers screened through schools completed online consent forms for the assessment stage and the remaining background questionnaire and participants completed online assent forms at the beginning of the assessment. Participants were administered the two language tasks, the Block Design task and the SAT. These tasks formed part of a larger study which lasted approximately 90 min in total. Participants received £15 on completion of the assessment stage and any travel expenses were reimbursed. Entry into a prize draw to win a £50 shopping voucher was offered as a reward to complete the screening questionnaires. Brief reports of individuals’ results were sent to parents/carers and findings from the overall study were shared with parents/carers in the form of a newsletter.

### Statistical Analysis

Stata 14 (StataCorp., 2015) was used to analyse the data. Instead of analysing language as a continuous construct, participants were categorised into groups of DLD and TLD status for two reasons. Firstly, children with DLD have disordered language development, not simply a delay, with the majority of the literature investigating DLD and associated socioemotional difficulties examining DLD as an entity based on a clinical cut-off and parental report of poor language functioning (Bishop et al., 2016). Secondly, previous research has suggested an absence of a linear relationship between language ability and severity of socioemotional problems (Fujiki et al., 2002; Hart et al., 2004), therefore analysing language ability as a continuous scale was not deemed useful. Following tests for assumptions, chi squares and ordered logistic regression were used to analyse group differences in the demographic variables. The variables of age and IDACI Rank were significantly skewed and therefore transformed before running regression analyses. Measures of Spatial Reasoning (Block Design subtest) and Receptive Language (Word Classes—Receptive subtest) were also significantly skewed and transformed before regression analysis. In each case, the *ladder* function in Stata was used to determine the most appropriate transformation. Group differences in the SDQ subscales of Peer Problems and Emotional Problems were analysed using negative binomial regression due to the most frequent responses being zero. Age, sex and IDACI Rank were entered as covariates in all analyses. SAT responses were transcribed by the first author. Author 1 and a second rater who was blind to group status (author 2) coded the transcripts in a random order, following an adapted version of Klin’s (2000) protocol obtained from the University of Cambridge. When raters were unsure or when large discrepancies in ratings were encountered, the raters convened and discussed the scores until agreement was reached. Intra-class correlations (ICC) were calculated to determine inter-rater reliability. A two-way mixed effects model was used to calculate intra-class correlations, treating the rater as a fixed effect and index as random effects, as each index was scored by the same set of raters. Consistency of agreement (CA-ICC) was used to determine whether scores differed by the same constant value for all the targets, as recommended by McGraw and Wong (1996) when the rater is random. Table 2 shows the intra-class correlations for each of the indices. Inter-rater reliability coefficients ranged from good (.8) to excellent (.9) (Cicchetti, 1994). Scores from the ‘blind’ rater (author 2) were used for analysis. The number of words, T-Units, ToM—Affective Index and ToM—Cognitive Index were transformed to account for the highly skewed data and independent t-tests were used to analyse group differences. Group differences in the Animation Index and the Person Index were analysed using a Wilcoxon-Mann–Whitney (‘ranksum’) test to account for ordinal data. A Spearman’s correlation with Bonferroni correction was used to examine the relation between age and SAT performance. Hierarchical regression was then used to test the effect of SAT performance on the outcome of peer problems. Group status, age, sex and IDACI Rank were entered first, followed by the seven SAT indices.

**Table 2:** Intra-class correlations for SAT scoring

SAT Index	*r*
Number of T-units	.95
Animation Index	.87
Person Index	.96
Salience Index	.91
ToM—Affective Index	.84
ToM—Cognitive Index	.93

## Results

### Group Differences in Cognitive, Language and Socioemotional Measures

Table 3 illustrates that there was a significant group difference on all cognitive, language and socioemotional measures. The DLD group scored significantly lower on the Block Design subtest; however, it should be noted that the overall mean score for the DLD group was still within the normal range for the Block Design subtest (*M* = 10, *SD* = 3). As expected, the DLD group performed significantly worse than the TLD group on the CELF-4 Recalling Sentences subtest which provides a measure of expressive language, and on the CELF-4 Word Classes—Receptive subtest which provides a measure of receptive language. Parent-ratings of emotional and peer problems were significantly higher for the DLD group compared to the TLD group.

**Table 3:** Mean (SD) scaled scores from cognitive, language and socioemotional tasks for the Developmental Language Disorder (DLD) group and Typical Language Development (TLD) group

	DLD (n = 26)	TLD (n = 27)	DLD vs TLD	Cohen’s d [95% CI]
Spatial reasoning^a^	8.15 (2.84)	11.89 (2.47)	− 67.64 (− 100.68, − 34.60)***	1.41 [.80, 2.00]
Expressive language^b^	4.92 (2.92)	9.93 (2.96)	− 4.71 (− 6.53, − 2.89)***	1.70 [1.06, 2.32]
Receptive language^c^	6.04 (2.92)	13.00 (2.39)	− 1.10 (− 1.40, − .80)***	2.62 [1.87, 3.35]
Emotional problems^d^	3.54 (.58)	1.63 (.40)	.92 (.30, 1.55)**	− .75 [− 1.30, − .20]
Peer problems^e^	3.31 (.50)	1.04 (.30)	1.04 (.34, 1.73)**	− 1.09 [− 1.67, − .51]

Statistics are *b* coefficients (95% confidence interval), controlling for age, sex and IDACI Rank. Regressions for Spatial Reasoning and Receptive Language are performed on transformed data

***p < .001; **p < .01

^a^Wechsler Intelligence Scale for Children—Fourth UK Edition (WISC-IV^UK^) Block Design subtest

^b^Clinical Evaluation of Language Fundamentals—Fourth UK Edition (CELF-4^UK^) Recalling Sentences subtest

^c^Clinical Evaluation of Language Fundamentals—Fourth UK Edition (CELF-4^UK^) Word Classes—Receptive subtest

^d^Strengths and Difficulties Questionnaire (SDQ) for parents—Emotional Problems subscale

^e^Strengths and Difficulties Questionnaire (SDQ) for parents—Peer Problems subscale

### Group Differences in SAT Performance

Table 4 shows the differences in mean ratings between the DLD group and TLD group for each of the SAT indices. As expected, the DLD group responded with a significantly lower number of words than the TLD group; however, the number of independent clauses (‘T-Units’) was not significantly different between the two groups. This is important to note as the ToM Affective and Cognitive Indices are calculated as a proportion of this number. Both groups scored similar levels of social attribution, with an average score of approximately 4 in the Animation Index. Scores range from 0 to 6 and are based on cut-offs that increase in complexity of social attribution. For example, the maximum score of 6 indicates the participant has mentioned at least one instance from each of the following categories: allusion to a person; higher level mental state terms expressing belief, thoughts, imagination, etc.; emotional terms resulting from a social situation (e.g. envious, jealous, sulking, etc.) or behaviours that are uniquely human by way of attempting to alter another character’s mental state (see S2 for details). A score of 4 corresponds to at least two instances of the above examples, but not two from the same category. The DLD group scored significantly worse on the Person Index compared to the TLD group, indicating that they made fewer mentions of psychological attributes, such as “bully” or “victim” (3 points) when asked to think of the shapes as people, and instead mentioned relative (2 points) or physical (1 point) properties. For example, participants in the DLD group were more likely to describe physical properties of the shapes (e.g. describing the big triangle as “a strong person”) or relative properties of the shapes (e.g. describing the big triangle as “a man” and the small triangle as “a girl”). The DLD group also scored significantly lower on the Salience Index, identifying approximately 43% of the twenty salient social features of the animation, compared to the 58% identified by the TLD group. For example, participants in the DLD group were more likely to describe the erratic movements of the small triangle as “bouncing around” instead of attributing emotions of fear or panic to the shape based on the storyline. In line with this, there was a significant group difference in the proportion of emotional mental state words used to describe the actions of the shapes, with the DLD group using fewer emotional mental state words than the TLD group. For example, participants in the DLD group were less likely to describe the big triangle as “angry” and the little triangle as “scared”.

**Table 4:** Comparison of mean scores (SE) on SAT Indices between Developmental Language Disorder (DLD) group and Typical Language Development (TLD) group

SAT Index	DLD (*n* = 24)	TLD (*n* = 27)	DLD vs. TLD
Number of words	209.50 (21.16)	321.52 (25.78)	3.67***
Number of T-units^a^	19.50 (1.90)	23.15 (1.54)	1.93
Animation Index (0–6)^b^	3.58 (.24)	3.93 (.21)	1.13
Person Index (0–9)^c^	7.04 (.37)	8.19 (.30)	3.10**
Salience Index (%)^d^	44.81 (3.04)	55.56 (2.76)	2.62*
ToM—Affective Index (%)^e^	.07 (.02)	.19 (.04)	2.42*
ToM—Cognitive Index (%)^f^	.30 (.04)	.40 (.08)	1.17

*ToM* theory of mind

*p < .05, **p < .01, ***p < .001

^a^Mean number of independent clauses in narratives 1–7

^b^Mean score from 0 to 6 based on level of social attribution in narratives 1–7

^c^Mean score from 0 to 9 based on psychological attributes given to shapes in narratives 8–10

^d^Proportion of social features identified out of a possible 20 in narratives 1–7 (n/20*100)

^e^Proportion of independent clauses containing emotional mental state words in narratives 1–7 (n/T-Units*100)

^f^Proportion of independent clauses containing cognitive mental state words in narratives 1–7 (n/T-Units*100)

Given the wide age range of the sample, a Spearman’s correlation with Bonferroni correction was run to examine the association between age and SAT performance. At the whole group level, there was a positive correlation between age and the score on the Salience Index (*r* = .39). Table 5 shows that, when separated by group, this association was only significant for the TLD group (*r* = .48) and not the DLD group. This suggests that older individuals in the TLD group were better at identifying key aspects of the story than their younger peers, but performance on the SAT did not vary by age for the DLD group.

**Table 5:** Spearman’s correlation of age and SAT indices, split by group (area above the diagonal denotes DLD group, italics below denotes TLD group)

	1	2	3	4	5	6	7	8
1. Age	–	0.27	0.14	− .002	0.23	0.35	0.17	0.07
2. Number of words	*0.27*	–	.92*	− .10	0.15	.39*	− .24	− .18
3. Number of T-units	*0.35*	.78*	–	− .13	0.12	.37*	− .31	− .23
4. Animation Index	*0.26*	.37*	*0.13*	–	.29	0.24	0.34	.63*
5. Person Index	*0.11*	*0.32*	.48*	*− .08*	–	0.11	0.17	.36*
6. Salience Index	.48*	.38*	*0.32*	*0.2*	*0.3*	–	0.17	0.29
7. ToM—Affective Index	*− .02*	*0.28*	*− .06*	.49*	*0.14*	*0.19*	–	.42*
8. ToM—Cognitive Index	*0.002*	*0.26*	*− .06*	.72*	*− .09*	*0.28*	.64*	–

*Correlation significant at the .006 level (Bonferroni adjusted)

### Relationship Between SAT Performance and Socioemotional Difficulties

Table 6 displays the output from the hierarchical regression used to analyse the influence of SAT performance on social and emotional difficulties. Demographic variables of group status, age, sex and IDACI Rank were entered in the first model. The second model included scores from the SAT indices (number of words, number of T-Units, Animation Index, Person Index, Salience Index, ToM—Affective Index and ToM—Cognitive Index). Finally, the third model added scores from the cognitive tests (AQ, Recalling Sentences subtest, Word Classes subtest and Block Design subtest). The first model was statistically significant (*F* (4,46) = 3.44, *p* = .02), explaining 16% of the variance in Peer Problems (adjusted R^2^). DLD group status was the only significant predictor. The second model was also statistically significant, explaining 20% of the variance (*F* (11,39) = 2.16, *p* = .04). However, the inclusion of SAT indices did not explain any additional variance (R^2^ Change = .15, *F* (7,39) = 1.32, *p* = .26) as DLD group status was again the only significant predictor. In the third model, performance on the ToM—Affective Index and scores on the AQ were significant predictors of Peer Problems and the overall model accounted for 45% of the variance (*F* (15,22) = 3.01, *p* = .01); however, the model did not significantly explain additional variance compared to the earlier models (R^2^ Change = .29, *F* (4,22) = .01, *p* = 1.00). Table 6 also shows hierarchical regression with emotional problems as the outcome. The first model significantly predicted 19% of the variance (*F* (4,46) = 3.96, *p* = .01), with both DLD group status and female sex identified as significant predictors. The inclusion of SAT indices accounted for 20% of the variance in the second model (*F* (11,39) = 2.16, *p* = .04), but did not explain any additional variance (R^2^ Change = .12, *F* (7,39) = 1.09, *p* = .38) as only DLD status remained a significant predictor of emotional problems. The third model was not a significant fit of the data (*F* (15,22) = 1.96, *p* = .07), although AQ score was a significant predictor of emotional problems.

**Table 6:** Predictors of socioemotional difficulties

Predictors	Peer problems			Emotional problems		
	Model 1	Model 2	Model 3	Model 1	Model 2	Model 3
Demographics						
DLD	1.91 (.61, 3.21)**	1.82 (.23, 3.42)*	.99 (− .91, 2.89)	2.05 (.55, 3.54)**	2.07 (.20, 3.93)*	1.93 (− .86, 4.72)
Age	0.01 (− .03, .05)	.01 (− .04, .05)	− .02 (− .07, .04)	− .03 (− .08, .01)	− .04 (− .09, .01)	− .06 (− .14, .02)
Sex	− .29 (− 1.54, .97)	0.20 (− 1.32, 1.71)	− .35 (− 1.94, 1.23)	1.51 (.07, 2.95)*	1.37 (− .39, 3.14)	1.94 (− .39, 4.26)
IDACI rank	− .01 (− .01, .01)	− .01 (− .01, .01)	− 3.98 (− .00, .00)	.01 (− .01, .01)	1.67 (− .01, .01)	.01 (− .01, .01)
SAT indices						
Number of words		.01 (− .01, .01)	− .01 (− .01, .01)		.01 (− .01, .02)	.01 (− .01, .02)
T-units		− .10 (− .25, .05)	− .04 (− .17, .09)		− .12 (− .29, .06)	− .08 (− .27, .11)
Animation		0.24 (− .47, .94)	.51 (− .22, 1.24)		.60 (− .23, 1.42)	.62 (− .46, 1.69)
Person		0.4 (− .01, .81)	.24 (− .14, .62)		.33 (− .15, .80)	.27 (− .28, .83)
Salience		0.01 (− .04, .06)	.02 (− .03, .07)		− .02 (− .08, .04)	.01 (− .07, .08)
ToM—affective		− 3.80 (− 9.52, 1.93)	− 6.19 (− 12.22, − .16)*		4.60 (− 11.28, 2.08)	− 6.11 (− 14.98, 2.76)
ToM—cognitive		0.06 (− 2.75, 2.86)	.76 (− 1.90, 3.42)		− .67 (− 3.94, 2.61)	.70 (− 3.21, 4.60)
Social, verbal and non-verbal measures						
AQ			.18 (− .48, .09)***			.16 (.03, .29)*
Expressive language			− .19 (− .48, .09)			− .26 (− .68, .17)
Receptive language			.25 (− .12, .62)			.50 (− .04, 1.05)
Block design			.24 (− .04, .51)			− .06 (− .45, .34)
R^2^	0.23*	0.38*	0.67**	0.26**	0.38*	0.57
Adjusted R^2^	0.16*	0.20*	0.45**	0.19**	0.20*	0.28
R^2^ change	–	0.15	0.29	–	0.12	0.19

B coefficients and 95% confidence intervals reported

*AQ* autism quotient

*p < .05, **p < .01, ***p < .001

### Relationship Between Language Ability and SAT Performance

In order to demonstrate that the SAT is appropriate for adolescents with DLD, the influence of expressive and receptive language ability on SAT performance was examined. Performance on the Person Index was significantly predicted by performance on the Recalling Sentences subtest in the DLD group (*b* = .35, [95% CI = .05, .66], *p* = .026), indicating that those with better expressive language ability were better at describing the shapes as people. To a lesser degree, expressive language ability predicted performance on the Animation Index in the DLD group (*b* = .22, [95% CI = .01, .44], *p* = .049). Additionally, performance on the Salience Index was predicted by performance on the Receptive subtest in the DLD group (*b* = 2.74, [95% CI = .40, 5.08], *p* = .024), indicating that better receptive language abilities were associated with a higher proportion of social features mentioned in the narrative. Receptive and expressive language abilities did not predict SAT performance in the TLD group.

## Discussion

The current study aimed to provide a better understanding of the role of social cognition in the socioemotional difficulties of adolescents with Developmental Language Disorder (DLD). Social cognition was measured by the Social Attribution Task (SAT; Klin, 2000), an engaging visual task that did not require strong receptive language skills to understand complex instructions. As expected, the DLD group demonstrated poorer social cognition skills by scoring significantly lower than the TLD group on three out of five indices on the SAT. Consistent with the literature, the DLD group received significantly higher ratings of peer and emotional problems compared to their peers; however, performance on the social cognition task was not a significant predictor of these socioemotional difficulties.

Previous investigations of the SAT have found that when presented with the simple, silent animation of basic shapes, typically developing children and neurotypical adults are likely to construct a story of the small circle protecting the little triangle from being victimised by the large triangle (Heider & Simmel, 1944; Klin, 2000). TD participants attribute social meaning to the story and human personalities and mental states to the shapes, demonstrating social cognition abilities. In the current study, the hypothesis that the DLD group would perform worse on the SAT compared to the TLD group was met to a certain extent. The DLD group scored significantly lower than their TLD peers on the Person, Salience and ToM—Affective Indices. These findings demonstrate that the adolescents with DLD were poorer at describing the shapes as people, despite being explicitly asked “What kind of person is the big/small triangle/circle?” Adolescents in the DLD group were more likely to view the shapes in terms of physical or relative properties instead of describing personality characteristics. This may reflect a difficulty in understanding the motivations for others’ behaviours. Additionally, the DLD group identified fewer key social aspects in their narrative of the story. There are twenty key points commonly mentioned by participants but the DLD group reported less than half of them when retelling the story. Furthermore, the DLD group used fewer emotional terms to describe the shapes compared to their TLD peers, in line with previous findings that children and adolescents with DLD are poorer than their TD peers at identifying emotions in faces and voices (Fujiki et al., 2008; Griffiths et al., 2020; Taylor et al., 2015). Overall, the poor performances on these SAT Indices are consistent with the literature that shows a deficit in social cognition abilities in children with DLD (Nilsson & de Lopez, 2016; Vissers & Koolen, 2016). Social interactions are full of nuanced cues that help us understand our conversational partners’ mental states and can aid in predicting their behaviour as well as how to respond appropriately. A difficulty describing personality traits, recognising social aspects of the story and identifying emotions on the SAT may indicate that adolescents with DLD have difficulty picking up cues in social interactions.

There were no other significant group differences in SAT performance, but the pattern of results was expected, with adolescents in the DLD group scoring lower than the TLD group on the Animation Index and the ToM—Cognitive Index. It should be noted that the Animation Index had the lowest rating of inter-rater reliability, but was still considered ‘good’ (Cicchetti, 1994). Both groups also used a similar number of cognitive mental state terms to describe the actions of the shapes. It is of interest that adolescents with DLD struggled with the affective but not the cognitive elements of theory of mind, suggesting that they had more difficulty with labelling emotions than with identifying wants or beliefs. This could be due to the more abstract linguistic nature of emotions. As expected, the DLD group used significantly fewer words than the TLD group to describe the video; however, the two groups’ responses consisted of a similar frequency of independent clauses (T-Units). This demonstrates that the two groups gave answers with a similar number of complete thoughts but the DLD group’s sentences were considerably shorter. The lack of a group difference in independent clauses is important to note given that the ToM Indices were calculated based on proportion of T-Units; therefore, there was no bias in scores due to the DLD group’s natural tendency to give shorter answers.

As social cognition is still developing in adolescence, and given the wide age range of the sample, we expected that there may be an effect of age on SAT performance. Age was significantly associated with the Salience Index, but when separated by group this effect only remained for the TLD group. The SAT performance in the DLD group did not vary by age, suggesting the social cognition difficulties were consistent within this group. This finding highlights the need to provide adolescents with DLD support to understand and process social cues because these difficulties are not necessarily ameliorated by age.

We next examined whether performance on these specific SAT indices predicted socioemotional difficulties. In the current sample, higher ratings of peer and emotional problems were reported by the parents of the DLD group compared to the TLD group, consistent with the large body of evidence that indicates increased socioemotional difficulties in children and young people with DLD (Yew & O’Kearney, 2013). However, when SAT indices were added to the model there was no significant change. This is in contrast to the previous literature demonstrating the link between poor social cognition abilities and social problems in the DLD population (Andres-Roqueta et al., 2016; Bakopoulou & Dockrell, 2016; Botting & Conti-Ramsden, 2008). Of course, as noted previously, social cognition is an umbrella term and the tasks used in the previous literature do not measure the same aspects of social cognition as the SAT.

Despite the lack of association between social cognition and socioemotional difficulties the current study still presents some interesting findings. Expressive language predicted scores on the Person Index and, to a lesser extent, the Animation Index, while receptive language predicted scores on the Salience Index. This association suggests that language skills can explain some of the poorer social cognition abilities in the DLD group. Adolescents with DLD who had poorer language skills had lower scores on select SAT indices than their peers with stronger language skills. Interestingly, this effect was not found in the TLD group, suggesting that for adolescents in the DLD group their language skills were directly related to their understanding of certain social cues. Critically, the lack of difference in T-Units suggests that the DLD group did not simply have a difficulty with verbalizing responses, as the narratives of both groups consisted of a similar number of utterances. Instead, this may reflect a deeper association between the specific language skills needed for social cognition. Of course, given the small sample size, it is difficult to fully resolve the question of whether SAT performance results from poorer awareness and understanding of others’ mental states or from poorer vocabulary. Nevertheless, these findings add further support to Vissen and Koolen’s (2016) second causal model explaining the relation between language and ToM and to the wider literature, such as the alexithymia language hypothesis which implicates language difficulties as casual in difficulties identifying emotions (Hobson et al., 2019).

It is important to note, however, that only three of the SAT indices were related to language ability. Therefore, further exploration of the causal mechanisms between language, social cognition, and indeed socioemotional difficulties in adolescents with DLD is necessary. Indeed, the findings from this study also warrant further examination because the SAT reflects the dynamic nature of social relationships and the interpretive skills that are essential during interactions in everyday life. For example, rather than relying on frequency data the Animation Index measures the complexity of social interpretation through the levels of social attribution in different categories, providing more detail about varying degrees of understanding than a ‘pass/fail’ measure (Klin, 2000). Further exploration of the SAT within the DLD population is encouraged.

Nevertheless, the current study is not without its limitations. Recruiting adolescents with DLD but without a diagnosis of ASD resulted in a small sample size and reduced statistical power. The size of the DLD group was increased by the use of parent report of historical language difficulties, which were confirmed by poor performance on the expressive and receptive language subtests. Still, the conclusions from this study should be interpreted with caution and a larger follow up study would be beneficial. Inclusion of a language-age-matched control group may help to further clarify the contribution of language and whether poor social cognition abilities are a deficit or a delay in individuals with DLD. Perhaps inclusion of pragmatic language assessments, such as the Making Inferences or Conversational Skills subtests in the CELF-5 (Wiig et al., 2013) may provide more information about the association with different language skills more suited to social interpretation. Furthermore, the SAT can only provide a measure of an individual’s ability to interpret others’ actions, not a measure of how the adolescent themselves would interact in a social situation. To address this, observational studies of social interactions among adolescents with DLD could be explored using an adolescent equivalent of the Manchester Inventory for Playground Observation (MIPO) (Gibson et al., 2011). Alternatively, measures such as eye-tracking or virtual reality could examine how participants interpret social cues directed towards themselves. For example, a study employing eye-tracking techniques found that children with DLD were more similar to their TD peers than an autistic comparison group who avoided looking at speakers’ faces during a social interaction (Hosozawa et al., 2012). Future studies could replicate this method with adolescents with DLD. Another avenue for exploration is measuring physiological responses to social interactions, using a tool such as salivary samples of cortisol levels to measure stress, which could provide more nuanced findings of how adolescents with DLD interpret and react to social situations.

## Conclusion

A proficiency in social cognition is extremely beneficial for communicating with others: Being able to ascribe intent and identify others’ emotions allows one to interpret others’ motivations and predict future actions, leading to more successful social interactions. A deficit in social cognition could explain the increased peer problems seen in adolescents with DLD and could aid in understanding how to improve these social difficulties. The current paper found that adolescents with DLD perform significantly worse than their TLD peers on selected indices of an animated social cognition task. However, social cognition abilities did not account for the variance in parent-rated peer or emotional problems. While the preliminary results from this task are interesting, further research with a larger sample size and additional language measures is advised to determine the effect of language ability on social cognition abilities.

### Supplementary Information

Below is the link to the electronic supplementary material.

Supplementary file1 (DOCX 21 kb)

Supplementary file2 (DOCX 19 kb)
