# Supplementary material for: Social Cognition in Adolescents with Developmental Language Disorder (DLD): Evidence from the Social Attribution Task
Source: J Autism Dev Disord. 2022 Aug 15;53(11):4243–57. doi: 10.1007/s10803-022-05698-6 (PMC10539456; doi:10.1007/s10803-022-05698-6)
Supplement: Supplementary file 1 — Supplementary file1 (DOCX 21 kb) [file 10803_2022_5698_MOESM1_ESM.docx]

Social cognition in adolescents with Developmental Language Disorder (DLD): Evidence from the Social Attribution Task

**Autism and Developmental Disorders**

**Claire L Forrest, Vanessa Lloyd-Esenkaya, Jenny L Gibson, Michelle C St Clair**

**Claire Forrest, IOE UCL’s Faculty of Education and Society, University College London, Claire.forrest@ucl.ac.uk**

# **S1. Scoring template for the Social Attribution Task (SAT).**

## S1.1 Scoring T-Units

A T-Unit is an independent clause, plus all clauses dependent on it. Every independent clause (plus its dependents) equals 1 T-Unit and should be recorded on its own line.

T-Units are supposed to be the smallest grammatically meaningful units of an utterance, so a good test of whether to include something as a part of a T-Unit, or to start a new one, is to ask yourself “does it make sense by itself?”

Clauses joined by co-ordinating conjunctions (such as *and, but, so*) are usually considered as separate T-units, as long as each element can stand alone. E.g. *They’re playing and the big triangle opens the door and now they’re running behind* = 3 T-Units

*They’re playing* (T1) *and the big triangle opens the door* (T2) *and now they’re running behind* (T3)

You’ll often see in the narratives that children start with “and” at the beginning of a new T-unit. However, sometimes a clause containing “and” will be counted as a single T-Unit. E.g.

*He tried and tried* = 1 T-Unit

*He goes straight through the house and breaks it apart* = 1 T-Unit

In the above examples, the subject of the second element of the clause is omitted but can be linked to the subject of the first element – in both cases here the pronoun “he.” Therefore, a single T-Unit is counted. Note that the second elements (*and tried*; *breaks it apart*) do not pass the “does it make sense by itself?” test, so this gives another clue.

Dependent clauses or elements may tell you something about the timing (while, after) or characteristics.

*That one’s hiding while the triangle’s going for the other kid* = 1 T-Unit

because “*while the triangle’s going for the other kid”* is a dependent of “*That one’s hiding”*

## S1.2 Salience Index (SI) (Narratives 1-7)

This index examines the information content of the child’s narrative. Clearly, there is no need for explicit use of the words describing the elements of the social story; **the element is scored as present or absent in terms of whether or not the idea is represented, explicitly or implied**, in the participant’s narratives. Work through the narrative to determine which of the story elements are present. Provide evidence (in quotation marks) from the child’s narrative in the space below and the number of points (0 or 1) awarded for that item. For example, for item 1, if the child refers to the rectangle as a ‘house’, then their narrative should be reproduced below (e.g., “they went into the house”) and a score of 1 awarded.

| 1.Rectangle is human enclosure |
| --- |
| 2.Recognition of three actors (rectangle not an actor, 3 agents throughout) |
| 3.Little triangle and circle are together (may be implicit) |
| 4.The big triangle and the small triangle fight |
| 5.Indication of the direction of hostility: The big triangle is the aggressor, the little triangle is resistant |
| 6.The little triangle is overwhelmed by the big triangle (e.g. the big triangle wins, the big triangle scares off the little triangle) |
| 7.The little circle tries to avoid conflict (e.g. hides, cowers, seeks protection) |
| 8.The big triangle searches for the little circle (e.g. entraps, tries to catch) |
| 9.The little circle panics (e.g. is afraid, scared, terrified) |
| 10.Indication that the little triangle came to the little circle’s aid (e.g. save, rescue,  help) |
| 11.The little circle escapes the big triangle (e.g. evades, flees, gets away from) |
| 12.The big triangle is trapped inside the enclosure |
| 13.The little circle and the little triangle celebrate (e.g. are happy, dance, rejoice) |
| 14.Proposition explaining the reason for celebration (e.g. escaped from the big  triangle, are free) |
| 15.Indication that the big triangle chases the little triangle and the little circle (e.g.  goes after, pursues them) |
| 16.Indication that the big triangle momentarily does not know where the little triangle  and circle are (as a result of the big triangle’s momentary search of the other two  shapes inside the rectangle) |
| 17.The little triangle and the little circle are successful at evading the big triangle (e.g.  they escape, run away) |
| 18.The big triangle is frustrated (e.g. mad, angry) |
| 19.Proposition of explanation for the big triangle’s anger (e.g. because he failed to  catch them) |
| 20.The big triangle breaks the enclosure |

**Scoring**.

(Number of elements of the story included in the participant’s narration / 20) *100

## S1.3 Animation Index (AI) (Narratives 1-7)

This index captures the level of sophistication of social attributions made in the narratives. It includes allusions to behaviours, perceptions, emotions, cognition, relationships, and words denoting (explicitly) a symbolic representation. The categories are not scored; the levels of attribution in each category are (A through M).

| 1. **Behaviours (doing something):** |
| --- |
| 1. Behaviours which necessitate actors or agents, but which are not uniquely or necessarily human behaviours, nor do they necessarily require any attribution of mental or feeling states (**e.g., chasing, fighting, destroying, but NOT ‘go’ verbs)** [code = 1a] |
| 1. Verbs or behaviours which do not involve an explicit mental state but are uniquely human **(e.g., talking, says, or a quotation)** [code = 1b] |
| 1. Behaviours which are uniquely human by virtue of implied indication of a shared mental state without which the behaviour cannot occur (**e.g., cheering, celebrating, trapping, hiding, dancing around, playing**) [code = 1c] |
| 1. Behaviours which are uniquely human by virtue of direct indication of an awareness by one character of another’s mental state, accompanied by an attempt to alter the second character’s mental state **(e.g., intimidation, deception, trickery, bullying, arguing, joking, rebuffing, taunting, helping)** [code = 1d] |
| **2. Perceptions:** |
| 1. Sensory experiences or attention which are not uniquely human **(e.g., look, watch, see, notice)** [code = 2e] |
| **3. Emotions (feeling something):** |
| 1. Emotional terms that usually result from a behaviour or an action, but which do not necessarily result from a social action, or which are not uniquely human **(e.g., happy, sad, scared, mad, alarmed, panicked)** [code = 3f] |
| 1. Emotional terms which result only from a social situation **(e.g., envious, jealous, sulking, bitter, mended his ways, expressing sour grapes, annoyed, admiration)** [code = 3g] |
| **4. Cognition, intention, motivation (usually thinking something):** |
| 1. Lower developmental level, mental state terms expressing desire or knowledge **(e.g., want to, know, mistake)** [code = 4h] |
| 1. Higher developmental level, mental state terms expressing belief, thoughts, imagination, plans (**e.g., pretending, remembering, decision, trying to**) [code = 4i] |
| **5. Relationships or personality traits:** |
| 1. Allusion to a person as constrained by his or her features **(e.g., big guy, little guy, kid)** [code = 5j] |
| 1. Allusion to a person as constrained by his or her relationship to another **(e.g., is a daddy, mummy, or baby)** [code = 5k] |
| 1. Allusion to a person as constrained by his or her actions or attribution of personality traits **(e.g., to be a bully, friends, companions, curious, timid, shy)** [code = 5l] |
| **6. Symbolic nature:** |
| 1. An acknowledgement of the symbolic nature of an object or shape (e.g., represents, stands for, symbolizes, a home, domain) [code = 6m] |

**Scoring procedure.**

*Score Criteria*

0 No human agency; mechanistic; geometric reasoning only

1 A or E or J

2 B or C of F or H or K or M

3 D or G or I or L

4 At least two of D or G or I or L, but not two of the same category

5 At least three of D or G or I or L, but not two of the same category

6 Four of D or G or I or L, but at least one of each

## S1.4 Theory of Mind Index (Narratives 1-7)

### S1.4.1 Cognitive mental states (CogA, CogB, CogC, CogD).

This index examines the use of cognitive mental state terms in narratives. Cognitive mental states are defined as:

1. Terms expressing one characters’ desire or knowledge **(e.g. he wants, she knows)**
2. Behaviours which not only implicitly indicate a shared cognition, thought, or plan between two characters but which cannot exist without it **(e.g., trapping, rescuing, sneaking, hiding, spying)**
3. Terms expressing one character’s belief, thought, imagination, intention, plan, motivation **(she thinks, she is planning, he’s pretending that)**
4. Behaviours which explicitly indicate a shared cognition, thought, or plan between two characters in which one character intentionally impacts on the other’s cognitive state **(e.g., intimidation, deception, trickery, bullying, arguing, joking, rebuffing)**

### S1.4.2 Affective mental states (AffA, AffB, AffC).

This index examines the use of affective mental state terms in narratives. Affective mental states are defined as:

1. Emotional terms that may not be the result of social interaction or may not be uniquely human
2. Behaviours which not only implicitly indicate a shared emotional state between two characters but which cannot exist without it **(e.g., cheering, celebrating, hugging, high-fiving)**
3. Emotional terms which result only from a social situation **(e.g., envious, jealous, sulking, bitter, mended his ways, expressing sour grapes, admiration)**

**Scoring procedure.**

Sum Cognitive Mental States (SumCog) = CogA + CogB + CogC; SumCog/No. T Units

Sum Affective Mental States (SumAff) = AffA + AffB + AffC; SumAff/No. T Units

## S1. 5 Person Index (PI) (Narratives 8-10)

Here, children are instructed explicitly to perceive the shapes as people. This index measures the participant’s ability to derive personality features from the shapes’ actions in the animation. Children’s responses are coded according to their level of sophistication. These involve, in increasing level of sophistication:

0 = nothing (no response, triangle, circle, behaviour)

1 = purely physical properties: descriptions based on the shapes’ form, e.g., big, small, skinny

2 = relative properties: descriptions of the interrelated social (e.g., family) roles of the characters although still related to their relative shape, e.g., adult, dad, mother, grown-up, kid, boy, baby

3 = psychologically-derived features: these attributions reflect characterological statements, e.g., curious, timid, bully, mean, shy, naughty, i.e., features that the characters would carry with them beyond the specific events portrayed in the video.

**Scoring procedure.**

The description of each figure (big triangle, small triangle, and circle) should be scored according to the level of sophistication. Scores should then be summed to yield a total Person Index ranging from 0 to 9.

Note: If there is a misattribution (i.e. psychologically-derived feature for the wrong shape) the maximum score is 2 for that shape.
