# Supplementary material for: Social Cognition in Adolescents with Developmental Language Disorder (DLD): Evidence from the Social Attribution Task
Source: J Autism Dev Disord. 2022 Aug 15;53(11):4243–57. doi: 10.1007/s10803-022-05698-6 (PMC10539456; doi:10.1007/s10803-022-05698-6)
Supplement: Supplementary file 2 — Supplementary file2 (DOCX 19 kb) [file 10803_2022_5698_MOESM2_ESM.docx]

Social cognition in adolescents with Developmental Language Disorder (DLD): Evidence from the Social Attribution Task

**Autism and Developmental Disorders**

**Claire L Forrest, Vanessa Lloyd-Esenkaya, Jenny L Gibson, Michelle C St Clair**

**Claire Forrest, IOE UCL’s Faculty of Education and Society, University College London, Claire.forrest@ucl.ac.uk**

# S2. Examples of SAT narratives from participants randomly selected from each group.

## S2.1 Example from Participant in DLD Group

### S2.1.1 Narrative 1 “What happened in the video clip?”

Well I thought, well it was just a normal triangle, being just walking around you know. Then a smaller triangle trying to chase a circle was like going round the screen and like. Then the big triangle was opening the door to like stay away. And then like the circle went in. The big triangle and the smaller triangle were having a fight. Whilst the smaller triangle got himself stuck in the…like box...thing. The big triangle had him cornered. But the circle moved around everywhere. And then um the circle got out and they were both being chased by the big triangle and they ran off and the big triangle just destroyed everything.

### S2.1.2 Narratives 2-7 “What happened here?”

**1^st^ clip**.

Well the big triangle just obviously seemed to have redone his built his box so he was inside just close the door up, then the small triangle and the circle came along they’re friends. He noticed and he opened the door then…. the small triangle and the circle just stood there.

**2^nd^ clip**.

He um…well the big triangle was like talking to them I think. Then he ??? prepared to attack the small one the small triangle. Then he attacked then the circle ran off. Got himself stuck in the doorway again I think. And then he closed the door and the big triangle and the small triangle kept on fighting each other.

**3^rd^ clip**.

Circle got trapped again, triangle was going to attack him but then the small triangle was going to attempt to open the door.

**4^th^ clip.**

The circle escaped by jiggling around the big triangle gets trapped in there the triangle circle circle around each other.

**5^th^ clip*.***

Uhmmm…well they’re happy and the big triangle gets out starts chasing them around the building around around around around ??? and eventually they fly off again into the distance.

**6^th^ clip.**

The big triangle twists around smashes through the door and breaks the entire thing.

### S2.1.3 Narratives 8-10 “What kind of person is the big triangle/little triangle/circle?”

**Big triangle.**

Bully.

**Little triangle.**

A person who’s trying to help the circle.

**Little circle.**

The smallest one, the one that’s more afraid and the one that’s being bullied.

## S2.2 Example from Participant in TLD Group

### S2.2.1 Narrative 1 “What happened in the video clip?”

So there’s a big triangle in the rectangle and it tries to get out. And then it gets out. And then a little triangle and a small circle comes… in. and then it bounces around the little circle. And then the big triangle comes and like bounces into the little triangle and then the little circle goes up to the rectangle and like, the side opens and then it goes inside and then gets trapped in there. But then the big triangle… barges into the side and it opens and then the little circle comes out and the big triangle goes in. and then the side closes. And then it opens again and the ball goes in, but then it closes again. And then the little triangle barges into the side and it opens. And then the… little circle comes out and then…barges into the small triangle and bounces into it. And then the big triangle comes out of the rectangle and chases the little triangle and the little circle around the rectangle. And then the big triangle goes back into the rectangle and then the little triangle and the little circle goes off the screen.

### S2.2.2 Narratives 2-7 “What happened here?”

**1st clip.**

The big triangle barged into the side of the rectangle – the inside of the rectangle. And it opened and then it closed again. And it was in there. Then the little circle came in… onto the screen. And then the little triangle bounced around the little circle.

**2^nd^ clip.**

The big triangle came out of the rectangle and saw the little triangle and then barged into the little triangle like bounced, into it. While the little circle went up to the rectangle which was open and it …there was a little gap in between the kind of the door bit of the rectangle, trying to close but the ball, the circle got stuck. And then it moved a bit and then it got pushed inside by the door bit of the rectangle.

**3^rd^ clip.**

The little circle was bouncing around in the rectangle and then the big triangle opened up the rectangle and like, pushed it down really far. And then it went into the rectangle and then it closed and then the little circle bounced really, really quickly around the bottom of the rectangle and the little triangle went into the side of the rectangle but didn’t get in.

**4^th^ clip.**

The little triangle - wait, the little circle and the big triangle were still in the rectangle and then little triangle opened up the rectangle and the ball came out, and then it shut again trapping the big triangle inside and then the little circle and the little triangle bounced into each other and around each other.

**5^th^ clip.**

The big triangle came out and the rectangle stayed open and then the big triangle chased the little triangle and the little circle around and around the rectangle and then the little triangle and the little circle went off the screen and the triangle went inside the rectangle and came back out again.

**6^th^ clip.**

The big triangle… shut the rectangle and then it went back towards the side of the screen and then charged into the side of the rectangle and split it into 4 pieces and then it went inside but wasn’t inside, it was just, blown apart.

### S2.2.3 Narratives 8-10 “What kind of person is the big triangle/little triangle/ circle?”

**Big Triangle.**

Bit of a bully.

**Little Triangle.**

Um…quite an innocent little person.

**Little Circle**.

Maybe the little triangle’s friend? Like … stayed with him.
